# Content validity of the Profiles of Early Expressive Phonological Skills-Brazilian Portuguese (PEEPS-BP) - Expanded List

**DOI:** 10.1590/2317-1782/20232022083en

**Published:** 2024-02-02

**Authors:** Simone Nicolini de Simoni, Denis Altieri de Oliveira Morares, Karina Carlesso Pagliarin, Márcia Keske-Soares

**Affiliations:** 1 Departamento de Fonoaudiologia, Universidade Federal de Santa Maria - UFSM - Santa Maria (RS), Brasil.

**Keywords:** Child, Language, Speech Test, Vocabulary Test, Validity

## Abstract

**Purpose:**

To carry out the cross-cultural adaptation of the Instrument Profiles of Early Expressive Phonological Skills- Brazilian Portuguese (PEEPS-BP) - Expanded list, performing content validation.

**Methods:**

Cross-cultural, quantitative and cross- sectional adaptation study, considering psychometric criteria. A study was carried out on the list of 423 words from the Communicative Development Inventory - MacArthur - Words and Sentences, adapted to Brazilian Portuguese. The method was divided into four steps. The list was judged by expert judges (JE) and non-specialist judges (JNE), considering as a criterion the familiarity of the word for a child aged between 24 and 36 months, and the representativeness of the word with toy/object, contemplating Steps 1 and 2. The child judges analyzed, in a data collection situation, the familiarity and representativeness of the stimulus-words, presented in Step 3. Afterwards, the pilot study - Step 4, was carried out with the selected stimulus-words. In the statistical analysis by expert and non-specialist judges, the Fleiss’ Kappa and Gwet Concordance index was used. In the analysis of the responses of the child judges and in the Pilot Study, the analysis was made in relation to the type of response of the child, specifically scoring the spontaneous naming of the toy/object, scoring qualitatively.

**Results:**

The result of Steps 1 and 2, and the agreement of the statistical tests for the Familiarity and Representativeness criteria was 45.7% for JE and 76.4% for JNE, and a result of 100% for the agreement of Representativeness. A total of 122 words were analyzed, resulting from previously established criteria, totaling 34 words (exclusion of one word by the researchers), totaling 33 stimulus-words. In Stage 3, of the 33 stimulus-words applied, nine presented spontaneous naming scores below expectations, being retested for Step 4, the Pilot Study. The result of the Pilot Study showed that of the nine retested stimulus-words, four of them still had a score below, being excluded from the study. Therefore, with the application of the Pilot Study, the expanded list of PEEPS-BP resulted in 29 words.

**Conclusion:**

The PEEPS-BP - Expanded List showed satisfactory evidence of content validity for the cross-cultural adaptation of the test.

## INTRODUCTION

The phonological and lexical assessment of children aged between 24 and 36 months is usually carried out using a sample of spontaneous speech. Thus, a significant amount of data is needed to obtain the phonological profile and vocabulary of a child in this age group. Therefore, for children older than three years, there are more options for standardized instruments available in the literature to meet this demand^(1-3)^.

The Instrument Profiles of Early Expressive Phonological Skills (PEEPS) was developed to assess the vocabulary and phonology of children aged between 18 and 36 months, referred to as PEEPS-US. The PEEPS-US^(3)^ has two lists for use in the assessment: the Basic List for children aged 18 to 24 months, consisting of 40 stimulus-words; and the Expanded List, to be applied together with the Basic List, in children from 24 to 36 months, which is composed of over 20 stimulus-words. The instrument in question, therefore, is dynamized with the use of toys that represent the stimulus words, and it is applied in a playful context.

Theoretical studies for the formation of PEEPS-US^(3)^ were carried out from existing words in the MacArthur-Bates Communicative Developmental Inventories^(4)^ (MacArthur-Bates - CDI), using the version of words and sentences, according to English age-acquisition norms. In this context, adapting an instrument validated in another country favors the exchange of scientific information, enabling cross-cultural studies and ensuring inferences and results that the test can provide for the population to be assisted^(5,6)^.

In Brazilian Portuguese, there are few instruments that provide the necessary components for language development in the same assessment, such as vocabulary and phonology, for example^(7,8)^. Furthermore, the evaluation in very young children, in the aforementioned age group, is also restricted in the national literature. Thus, it is worth highlighting that the instrument ABFW^(9)^, nationally recognized and a valuable contribution to Speech Therapy in Brazil, presents interconnected components in the same assessment, made available through pictures for the public from the age of two. Data referring to the validation of this instrument for the Phonology and Vocabulary Tests can be found in the doctoral research by authors Wertzner^(10)^ and Befi-Lopes^(11)^.

For the validation process of the phonology test, theoretical studies were carried out based on international speech assessment instruments, for the design of stimulus-words, with subsequent analysis by judges for the selection of illustration boards. The author presents normative data by age group, in addition to correlation analysis for the collection method of imitation and naming, distributing the results for the production of phonemes in the different groups studied^(10)^.

In the vocabulary test, the analysis of competence and lexical performance was carried out in nine conceptual fields, and also the comparison in children with and without speech/articulation disorders. The analysis presents normative data for the different classes of lexical designation processes, distributed among the researched sample^(11)^.

The cross-cultural adaptation of PEEPS-US3 to Brazilian Portuguese, with content validation from the Expanded List, entitled PEEPS-BP (Brazilian Portuguese) - Expanded List, aims to develop an instrument that will contribute to the early assessment of children, helping, above all, in diagnosis and clinical intervention, as well as in scientific research.

PEEPS-BP - Expanded List deals with the validation of the content of the stimulus words to compose the expanded list of the instrument in the national language, with criteria relevant to vocabulary and phonology, which is dynamized with children between 24 and 36 months. Therefore, this step corresponds to the process of verifying the suitability for the Brazilian reality and the linguistic analysis (semantic and phonological) of the items that compose PEEPS-BP - Expanded List. Thus, the content is verified based on the analysis of experts in the area, in order to understand the items that compose the instrument, pointing out its relevance and pertinence for its applicability in the Brazilian territory^(12,13,14,15)^.

The objective of this study sought evidence of content validity of PEEPS-BP - Expanded List, to be applied along with the existing Basic List in children aged 24 to 36 months.

## METHODS

This study is a cross-cultural, quantitative and cross-sectional adaptation, considering psychometric criteria. The adaptability of PEEPS-BP was authorized by PEEPS-US authors and elaborated together with the authors of the PEEPS-BP Basic List^(16)^ that preceded the application of the Expanded List that is the focus of this article.

This research was duly approved by the Research Ethics Committee, registered under number 18419319300005346. Authorization was requested from all participants involved, in accordance with the rules of the National Health Council - Resolution 466/12. All participants were required to sign the Free Informed Consent Form (FICF). In addition, the children included in the study assented orally to their participation in the research.

### Participants and procedures

The sample consisted of different participants, who were recruited for convenience, and/or contacted by the authors via e-mail and/or telephone. This audience was then recruited and composed one of the four steps of the adaptation process, namely: Expert Judges (Step 1), Non-Expert Judges (Step 2), Child Judges (Step 3), and Pilot Study (Step 4).

### Step 1 - expert judges

This step, initial in the process of adapting the instrument, aimed to verify which words are familiar to the vocabulary of a child aged 24 to 36 months by the analysis of the theoretical concepts by experts. Still, we sought to define, from these words, which could be represented by a toy. Therefore, professionals were contacted by email and invited to participate in this step of the research. All professionals who agreed to participate signed the FICF.

The sample of Expert Judges (JEs) was composed of nine professionals, including speech therapists and linguists with expertise in the area of language/speech, especially phonology and children’s vocabulary, all holding a doctoral degree.

In this step, each JE made his/her analysis individually for some two months, time available for them to send the answers. For analysis, a link was sent that directed the JE to two Google Docs forms. Both forms presented 423 words of the MacArthur Communicative Development Inventory of Brazilian Portuguese - words and sentences^(17)^.

In the first form, each JE should answer about the Familiarity with the word, that is, consider whether the word would be present in the receptive and expressive vocabulary of a child from 24 months. It should be indicated if, somehow, children in this age group would have already been exposed and would be familiar with the word. The judgment of the answers was carried out using the Likert Scale divided into: Extremely Familiar (1), Very Familiar (2), Familiar (3), Somewhat Familiar (4) and Not Familiar (5). The JE should classify, according to the Scale, the proximity of the word to the researched age group, judging, for example, words considered frequent in the vocabulary as Extremely Familiar.

In the second form, the JE responded to the criterion of Representativeness, that is, whether the analyzed word could be represented by means of a toy. In this form, the answer should be marked as “Yes” or “No”.

Once this step was completed, experts’ responses were organized into an Excel table for further statistical analysis.

### Step 2 - non-expert judges

This step has the same objectives and criteria as the previous one (Step 1). However, this group was composed of mothers of children between 24 and 36 months of age, who were called Non-Specialist Judges (JNE). All signed the FICF agreeing to participate in the research.

The JNE sample consisted of eight mothers who did not have technical/specific knowledge in the area of children’s language/speech and phonology/vocabulary, and who had children in the age group of the study. We sought to verify which words are present in the vocabulary of young children from the judicious look of those who live with infants on a daily basis.

In the same way as the JEs, the JNEs should respond to the two Google Docs forms, available in online format and with 423 words from the MacArthur Communicative Development Inventory of Brazilian Portuguese - words and sentences^(17)^.

The JNEs analysis also considered the same criteria for Familiarity and Representativeness. Regarding Familiarity, they were asked to assess whether the word belonged to the vocabulary of a child aged 24-36 months, indicating whether the word was: Extremely Familiar, (2) Very Familiar, (3) Familiar, (4) Somewhat Familiar, and (5) Not Familiar. Regarding Representativeness, the JNEs needed to point out whether they considered that the word could be represented by a toy, answering “Yes” or “No” .

As in Step 1, at the end the JNEs responses were organized in an Excel table, and submitted to statistical analysis. Therefore, both in Step 1 and in Step 2, the analysis was carried out across the statistical method, with the purpose of quantifying the concordance index between JEs and JNEs in relation to the accuracy of the words.

From this perspective, Fleiss’ Kappa - responsible for generating the discriminant function of the instrument data and the cutoff line of the judges' concordance in the 423 words - was used for the criterion of Familiarity, while the Gwet coefficient was used to analyze the criterion of Representativeness of words, considering the absolute value for this analysis. Afterwards, the sets were intersected to find words that had a higher concordance index in Familiarity and an absolute value in the Gwet coefficient for Representativeness.

In this scenario, the agreement index of 1 to 1.5 (17.8%) of the words was the first to be analyzed by the criterion, classifying them as “Extremely Familiar.” These words presented agreement at the intersection of Familiarity and Representativeness. This was considered the main cutoff point, totaling 42 words in the category.

Subsequently, the cutoff point was analyzed in the sequence between 1.5 - 2.5 to cover a greater number of words, which presented the same proposal of the intersection Familiarity and Representativeness. A total of more 200 words were included, which were added to the initial cutoff point of 1-1.5.

Finally, with the last analysis performed, it was decided to form the list of words for the research, presented in the results section, which comprised the cut-off point between 1 and 1.5-2. This cutoff point presents the words related to the intersection of Representativeness and agreement with the Familiarity criterion in the “Extremely familiar” and “Very familiar” axes.

Once this cutoff point was defined, the words were analyzed in correspondence from 1-1.5 to 2. A total of 122 words is present in this set, and they were those that obtained a significant and relevant score at the intersection between Familiarity and Representativeness. These 122 words were analyzed, still in Step 2, according to the following criteria:

- Words present in the original instrument that can be translated and adapted appropriately for BP;
- Priority for the first words obtained in the static analysis classification (42 words) that obtained the best score analyzed from the judges’ responses (<1.5), which are in the “Extremely Familiar” category.
- Words with syllabic structure formed by disyllables, followed by trisyllabic words that contemplate expected phonemes for the age of phonological acquisition of the study (24 to 36 months).

After completing Steps 1 and 2 of selecting the words to be inserted in the instrument, we proceeded to the content validation steps and application of the PEEPS-BP-Expanded List.

### Step 3 - child judges

Step 3 aimed to verify whether the selected words (Steps 1 and 2) were part of the vocabulary of young children, as well as whether the selected toys were suitable for recognition and spontaneous naming by children aged 24 to 36 months. To this end, the research authors purchased several commercially available toys, for the application of the instrument according to the final list of words from Steps 1 and 2.

The sample for this Step 3 consisted of four children selected for convenience, aged between 24 and 36 months, specifically two boys and girls females. The parents/guardians consented to participate in the research, and the children agreed orally to participate in the study.

In this step, speech-language evaluations were carried out to ensure the children’s cognitive, linguistic, auditory, and motor development as expected for the stage they were in. With this, only those who presented impairment in neurodevelopment, auditory system, oral, receptive and/or expressive language would be excluded from the research. With the results obtained, no participant was excluded.

The assessments described below were applied to each participant individually:

- General Anamnesis - an online interview was carried out using the Google Meet platform to gather information about the gestational period, pre-peri and post-natal complications, as well as factors related to child development, general health, everyday life situations, and social aspects related to health and family.
- Hearing Assessment - was carried out with the Transient Otoacoustic Emissions (TEOAE) device from the brand OTOREAD. In this exam, the child should present the answer “passed” in both ears, characterizing adequate cochlear function.
- Bayley Child Development Scale III (Screening)18 assesses three development domains: cognitive, linguistic (expressive and receptive communication), motor (broad and fine). The child should present a “competent” answer in the four levels of the scale according to their age group. It is important to emphasize that the researcher in charge has training for the application of this instrument.
- MacArthur Brazilian Portuguese Inventory - words and sentences^(17)^: parents/guardians should mark in the inventory protocol if the child produced at least 10 words of those provided in the document, that is, the child needed to express at least 10 words. It is an evaluative marker of the minimum level of speech production response.

Therefore, according to the applied tests, the four children were included in the research, characterizing neurotypical development for the selected age group.

The procedures in Step 3 were carried out in an appropriate room for data collection, that is, obtaining the children’s answers to the presented stimuli. Parents/guardians accompanied all stages of the research conducted with the child. In addition, data collection was recorded and filmed for later data analysis, a process with the parents’ consent and in accordance with the Research Confidentiality Term.

In a room, duly structured with children mats, the researcher made available the toys that represented the stimulus words, which were in a transparent bag for the child’s analysis. In the same toy, there may be the possibility of naming other words-stimuli of the instrument, such as, for example, the body parts of a doll. The instruction provided by the research was translated from the original language (English) to BP, composing the following sentence:

“Hello, I am going to show you some toys, you have to tell me the name of each toy and if you know them. In the end, you will be able to play freely with all the toys with your mommy/guardian.”

After the instruction, the child chose the toys according to their interest, since they were visible in the bag, and then they were encouraged to name it spontaneously, identifying the toy. Also, the original instrument allows the use of stimulus strategies as needed, which have to be performed using questions such as “What is this”? or “Tell Mommy the name of this toy,” as well as completing a sentence or using the repetition context.

In Step 3, the objective was just the spontaneous naming by the children based on the objects presented; therefore, the collection was conducted according to the child’s interest in manipulating the transparent bag. This form of application was later modified in Step 4 by separating the objectives into categories, in order not to disperse the child with all the toys exposed.

Data from this stage were analyzed, scoring if there was spontaneous naming by the child, or if they repeated the word provided by the researcher, or even if they did not know how to speak. Situations in which the child knew the word but was in doubt about the representativeness of the toy were also scored. In the meantime, in Step 3, the children’s answers were scored with 1 point for each word produced by the children in the “spontaneous naming” criterion and 0 point for “repetition or refusal” when speaking. Also, it was qualitatively analyzed when they presented reactions such as not knowing what the toy was or changing its name to another name, showing doubts about the representativeness of the item. The sum of the four children’s scores should be greater than three to consider the word as *adequate* for the representativeness of the toy and *adequate* for the Familiarity with the word.

### Step 4 - pilot study

In Step 4, Pilot Study (EP), the objective was to administer the PEEPS-BP- Expanded List in a real collection situation, based on the changes observed in Step 3.

For convenience, six children aged between 24 and 36 months were recruited for the EP, three boys and three girls. The parents/guardians agreed with the FICF and the children agreed orally to participate in the research. These children were submitted to the same instruments as in the previous stage (Stage 3) for inclusion in this stage of the study, that is, in order to confirm their child development within the expected parameters for their age group. Therefore, the procedures of general anamnesis, Auditory Assessment by TEOAE, Bayley Child Development Scale III (Screening)^(18)^ and assessment of the MacArthur Communicative Development Inventory of Brazilian Portuguese - words and sentences^(17)^ were carried out.

The stimulus words were represented by the toys and were previously selected according to the results of Step 3. From this, the objects were placed in smaller transparent bags and inside individual boxes, grouped according to the semantic category, or closest context, to favor interaction. The grouping of toys suited the categories of animals, kitchen utensils and food, dolls with body parts, and household utensils with vehicles, and toys and miscellaneous items.

When the researcher started applying the PEEPS-BP Instrument - Expanded List, she provided the initial instruction, translated from the PEEPS-US3 and adapted to the new PE collection format: “*I will show you the toys that are in these boxes, you have to tell me the name of each toy and if you know them. When we finish looking at all the boxes with the toys, you can play with all of them and the mother/father/guardian*.”

The toys, divided into the categories above, were given to the child, ensuring that they were randomly chosen, according to the original instrument. In this context and with the aim of helping spontaneous naming, when necessary, strategies were used with questions to facilitate the pronunciation of the word, such as “What is that”? or “Tell Mom the name of this toy,” as well as completing a sentence or its repetition. The application of the EP was recorded and filmed according to the criteria of the Term of Confidentiality for research data information collection and subsequent analysis.

In Step 4 (EP), the application of the PEEPS-BP-Expanded List Instrument was analyzed with the established format of toys arranged by semantic categories and a qualitative analysis of the modified copies was carried out in relation to the result of Step 3.

In the EP, the repetition of the word was not considered, only the spontaneous naming. Thus, the items tested should have a minimum score of five points to remain in the study, that is, the same criteria as in Step 3. Spontaneous naming considered throughout the study method concerns the identification and adequate recognition of the stimulus word in front of the object/toy, not being characterized by a repetition/model provided by the researcher. This issue implies considering in spontaneous naming possible omissions/phonological substitutions in stimulus-words, since the child’s phonological system is in a period of phonological acquisition and development. That is, in the age group studied, most children in typical acquisition did not acquire the CCV structure; therefore, if the child named “falda” [‘faw.da] for “fralda” [‘fraw.da] [diaper], it was considered that he/she had named the object spontaneously.

Chart 1 describes the methodological sequence used in the scientific research, indicating the participants and education level.

**Chart 1:** Methodological steps of the research

Content Validity	Sample number	Selection Criteria	Education of participants
Step 1-Expert Judges	9 Judges (6 Speech language pathologist and 3 Linguists)	Speech language pathologist emphasis on language/speech, especially phonological and vocabulary Linguist’s emphasis on phonology.	Postgraduate degree at doctoral level
Step 2- Non-specialist Judges	7 Judges	“Lay” mothers of children between 24 and 36 months, that is, without scientific theoretical knowledge about the development of aspects of oral language.	4 mothers with completed higher education
			3 mothers with completed high school
Step 3-Child Judges	4 children females (29 and 36 months) and males (27 and 31 months)	Children between 24 and 36 months, two males (27 and 31 months) and two females (29 and 36 months), typical cognitive development, broad and fine motor, expressive and comprehensive language. (According to evaluations carried out)	3 mothers with completed higher education
			1 mother with completed high school
			4 children enrolled in Nursery I
Step 4- Pilot Study	6 children females (32,33 and 34 months) and males (26,35 and 36 months)	Children between 24 and 36 months, three males and three females, typical cognitive development, broad and fine motor, expressive and comprehensive language. (According to evaluations carried out)	6 mothers with completed higher education
			3 children enrolled in Nursery I and II
			3 children not enrolled in schools.

## RESULTS

The results of Steps 1 and 2 culminated in 122 words, according to the applied agreement indices, result of the cutoff point of analysis of 1-1.5-2. According to the established criteria previously explained in the Method - Step 2 section, Items a-b-c, with regard to the 122 selected words, 34 words remained. However, one word was previously deleted: “*menino/menina*,” [boy/girl] due to the inflection of the morpheme that indicates the gender inflection *o/a*. Therefore, the result of Steps 1 and 2 totaled 33 words.

The result of the agreement between the JEs and JNEs, for the Fleiss’ Kappa in relation to the Gwet coefficient, presented a value equal to 45.7%, with a confidence interval of 95% for the JEs and 76.4% for the JNEs, with the same confidence interval considered. In relation only to the analysis of Representativeness, the Gwet concordance index, for JEs and JNEs, presented an absolute result of 100%.

It was observed that in Step 3, nine stimulus-words were qualitatively analyzed in relation to spontaneous naming and/or recognition of the toy had a lower score than expected in the application of the instrument for Child Judges. Therefore, it was decided to retest the toys/objects in Step 4.

In Step 4 of the EP, the results of the stimulus words analyzed qualitatively, and which still had a score lower than expected (five points) were excluded from the instrument. Thus, regarding the 33 words tested, the PEEPS - BP - Expanded List consisted of 29 stimulus-words for evaluating children aged 24 to 36 months.

The result of the application proposal was modified in Step 4 in relation to Step 3, without causing prejudice when compared to the original instrument, as it was still an analysis by the Child Judges for the recognition of stimuli. Therefore, the random delivery of toys was maintained, even if they were grouped into semantic categories. In view of this, the data collection scenario was satisfactory for the performance of the EP, in order to maintain the sequence for the other studies.

Chart 2 presents the sequence of the results by stages (Steps 1, 2, 3 and 4) of the stimulus words.

**Chart 2:** Results of Steps 1, 2, 3 and 4 for stimulus words

**STEP**	**NUMBER OF WORDS**	**WORDS**	**WORDS BELOW THE MEDIA**	**EXCLUDED WORDS**
**Step 1 and 2 - JE and JNE**	34 words	Banana, balloon, belly, mouth, doll, hair, truck, blanket, spoon, dog, bed, hat, cup, tooth, elephant, diaper, moon, lion, tongue, bottle, hand, sock, baby, ear, bird, foot, fish, leg, comb, juice, sun, soap, belly button, boy/girl	For this stage, words were not considered below average.	Boy/Girl
**Step 3 - Child**	33 words	Banana, Balloon, Belly, Mouth, Doll, Hair, Truck, Blanket, Spoon, Dog, Bed, Hat, Cup, Tooth, Elephant, Diaper, Moon, Lion, Tongue, Bottle, Hand, Sock, Baby, Ear, Bird, Foot, Fish, Leg, Comb, Juice, Sun, Soap, Belly button.	Truck, blanket, moon, sock, baby, bird, comb, leg and belly button. (Stimuli retested in Step 4)	At this stage, no words, were excluded.
**Step 4 - Pilot Study**	29 words	Balloon, banana, belly, mouth, doll, hair, dog, bed, hat, spoon, cup, tooth, elephant, diaper, lion, tongue, bottle, hand, ear, foot, fish, soap, sun, juice, truck, moon, sock, comb and leg.	For this stage, words were not considered below average.	Baby, blanket, bird and belly button

## DISCUSSION

Several steps are necessary to carry out the cross-cultural adaptation of an assessment instrument, with word content validation being the initial step in this process^(19)^. In this context, it is necessary to point out that the study of the words, the elements of the original instrument, and the choices of the judges/experts to participate in the process are decisive for the final content^(20,21)^.

In literature, the use of translation and back-translation is indicated as a possibility when carrying out the cross-cultural adaptation, but such practice was not possible to be followed in this study due to the fact that the acquisition of phonology, phonetic inventory, the vowel system and structures syllables of the English language are different from Brazilian Portuguese^(22,23)^. Even so, the same profile of the theoretical study presented in the original instrument was used, that is, the MacArthur Brazilian Portuguese Inventory - words and sentences^(17)^, already adapted for Brazilian Portuguese. This inventory comprises a list of words belonging to children’s vocabulary, so that it served as the basis for the selection of stimulus words. In addition, the choice of stimulus words, judges and the criteria involved were definitive for the proper elaboration of the expanded list, since these procedures help in validating the content for the adaptation of an instrument, as researchers in the area present in their studies^(24-26)^.

In addition, content validity comprises the assessment by expert judges in the area, who emphasized their theoretical and scientific knowledge about child development, as well as aspects of phonology and vocabulary, during the construction process of this study. This fact was also noted in relation to the non-specialist judges, who, despite not having theoretical mastery of the components of oral language, have daily contact with children aged 24-36 months. Based on academic studies, it is known that the acquisition of sounds and words depends partially on the environmental stimuli offered^(27)^, so this heterogeneity of non-specialist judges proved to be important for the judgment of stimulus-words, including different terms that are part of the interaction and social context of each interaction of the children^(28-30)^.

The judgment on the part of the children helped to verify the preliminary items of the instrument, the stimulus words, in addition to pointing out unsatisfactory toys for the representation of the item. In conducting the research procedure, it was observed qualitatively that the child often had the word in their vocabulary, but the object caused confusion or did not adequately represent what was being proposed. Therefore, the fact of always keeping the intersection in the statistical analysis between familiarity and representativeness of the stimulus word was a constant in the study, helping in the choice of the object/toy, reinforcing the choice of adequate words for the children’s vocabulary.

Phonological or vocabulary assessment mostly uses illustrative pictures as a content component, different from the proposal for the selection of stimulus words for this content^(9,31,32)^. This reality is different from what happens in the PEEPS-BP- Expanded List, since the toy to represent the word has to be judged by the non-specialist judge, even if it was judged previously by specialist judges. Still, child judges are essential for this type of validation, since this target population will be evaluated and benefited with the objectives of applying the instrument, and it is a criterion suggested in the psychometric part^(33)^. It is worth noting that the Instrument PEEPS-US and its adaptation are always applied using toys/objects.

Therefore, the evaluation by the judges involved in this process included content validation and its development with quantitative and qualitative procedures. Therefore, an instrument is valid in its content when it really evaluates the proposed objective, which in this case was to select familiar words for the vocabulary of a child aged 24-36 months and that were also represented by a toy. This practice was due to the methodology of the original instrument, which is carried out with concrete objects.

In view of this, the judges complied with the proposal of scoring the instrument stimuli, ensuring that the instrument represented adequately the objective of being evaluated. With the result of the statistics in relation to the familiarity with the word, theoretical criteria were established in order to understand the phonological component and the syllabic structure according to the acquisition of the age group of 24-36 months. However, as it is an instrument that assesses vocabulary and phonology concomitantly in a child population, some words that were included in the study showed the expected syllabic and phonological structure for mastery of acquisition after 36 months. This is justified by the fact that the word belongs to the children’s vocabulary, so that it is already known that the child could present some type of phonological omission, considered common for the age of acquisition.

Finally, this choice is justified because tests that involve evaluations need to include words -stimuli of varying complexity, as they provide varied and reliable answers about the child’s ability in situations outside their daily life^(13,14)^.

The PEEPS-BP- Expanded List presents stimulus-words suitable for the vocabulary of children aged 24-36 months, who met the criteria previously described in the Method. The use of these previously established criteria shows the effectiveness of the translation and cross-cultural adaptation of the instrument into Brazilian Portuguese, since the words with the best score were chosen, in different phonological contexts and syllabic structures.

The stimulus-words present both phonemes from the acquisition phase of the population studied, and phonemes that are acquired at a later age, performing varied phonological balancing and with different levels of complexity.

Given of this fact, the chosen words are justified for the PEEPS-BP instrument - Expanded List, which presents: in initial onset (OI) the phonemes /p/, /b/, /d/, /k/, /m/, /s/ e /ʃ/; in medial Onset (OM) the phonemes, /p/, /b/, /t/, /d/, /k/, /g/, /m/, /n/, /ɲ/, /ʃ/, /ɾ/, /l/, /x/, /ʎ/, allophone [tʃ]. In the medial coda position (CM), it presents the phonemes, /l/, /N/, and the archiphoneme /R/, and in the final coda position (CF) the phoneme /l/ and the archiphoneme /R/. For the Complex Onset (OC), it presents the segment, /fɾ/ em OI. The PEEPS-BP-Expanded List presents three monosyllable words, 16 disyllable words, seven trisyllable words, and three polysyllables.

The expanded list differs from the basic list, as it presents some phonemes not included in the basic list, and also presents segments of consonant acquisition considered late segments for the age in question assessed. That is, it presents components that the basic list did not have, valuing its application in children between 24-36 months, considering the vocabulary intended for them.

Furthermore, the application of the EP is characterized as a scale of procedures, materials and methods proposed in the application of the instrument, that is, it is a version of the complete study that involves everything that was foreseen in the methodology, in order to allow changes or improvements in the instrument in the phases that precede the investigation of criteria and constructs^(15)^. Thus, the importance of conducting a pilot study lies in the possibility of reviewing, testing, improving, and evaluating the collection scenario, as well as the instrument instructions and the research procedure. The EP is able to provide potential problems in the research, to be implemented before conducting the established sample number for the study.

Therefore, the EP presented here was administered to children with typical development in a real collection scenario, in order to be carried out as close as possible to the original instrument. With that, the children were randomly exposed to the toys, which were separated into the adopted semantic categories. In this context, the children had no difficulty in recognizing and producing the stimulus word from the toy, showing a favorable performance in terms of vocabulary and phonology. This format was established by division into semantic categories and for being randomly chosen by the child, according to the original instrument, as it was observed in Step 3 that they could disperse with the procedure adopted.

The cross-cultural adaptation with content validity fulfilled the psychometric requirements for the choice of stimulus words with the different judges adopted, reaching the objective of finding words familiar to children aged 24 to 36 months and that could be represented by a toy/ concrete object. Furthermore, the reinforcement of the application in a pilot study made it possible to identify failures and verify the applicability of the PEEPS-BP-Expanded List instrument.

In this sense, content validation as one of the relevant stages of cross-cultural adaptation presented the formation of the PEEPS-BP Expanded List for the Brazilian context. In order to follow other psychometric measures, it is still necessary to research the validation of criteria and normative data for the study population.

## CONCLUSION

The PEEPS-BP - Expanded List, adapted to Brazilian Portuguese, is considered an instrument for assessing infant vocabulary and phonology in children aged 24-36 months. Its cross-cultural adaptation presented word content validation, including analysis by different judges and, following the proposal of the original instrument, it will enable the assessment of young children. The evaluation of the PEEPS-BP considering its application in the early years of the child population will favor the follow-up and monitoring of the period of development destined to oral language, in aspects of vocabulary and phonology. The content validation presents the appropriate items for the Brazilian children’s reality, as well as the difference in the application of the instrument with objects/toys, favoring the interaction between professional and child, with the ludic element being employed at the evaluative moment.

The Instrument PEEPS-BP-Expanded can help in clinical speech therapy, as well as in research, in the monitoring of oral language development milestones and in the prevention and promotion of early childhood development milestones, with regard to aspects of expressiveness of phonology and vocabulary. It is known the importance of continuing the study, seeking normative criteria, from the established Expanded List.
