# Supplementary material for: Content validity of the Profiles of Early Expressive Phonological Skills-Brazilian Portuguese (PEEPS-BP) - Expanded List
Source: Codas. 2024 Feb 2;36(1):e20220083. doi: 10.1590/2317-1782/20232022083en (PMC10939398; doi:10.1590/2317-1782/20232022083en)
Supplement: Translation

# Translation: Validade de conteúdo do *Profiles of Early Expressive Phonological Skills-Brazilian Portuguese* (PEEPS-BP) - Lista Expandida

**DOI:** 10.1590/2317-1782/20232022083pt

**Authors:** Simone Nicolini de Simoni, Denis Altieri de Oliveira Morares, Karina Carlesso Pagliarin, Márcia Keske-Soares

**Affiliations:** 1 Departamento de Fonoaudiologia, Universidade Federal de Santa Maria - UFSM - Santa Maria (RS), Brasil.

**Keywords:** Criança, Linguagem, Teste de Fala, Teste de Vocabulário, Estudos de Validação

## Abstract

**Objetivo:**

Realizar a adaptação transcultural do Instrumento *Profiles of Early Expressive Phonological Skills- Brazilian Portuguese* (PEEPS-BP) - Lista expandida, realizando a validação de conteúdo.

**Método:**

Estudo de adaptação transcultural, quantitativo e transversal, considerando critérios psicométricos. Realizou-se o estudo da lista de 423 palavras do Inventário de Desenvolvimento Comunicativo- MacArthur- Palavras e Sentenças, adaptado para o Português Brasileiro. O método foi dividido em quatro Etapas. A lista foi julgada por juízes especialistas (JE) e juízes não especialistas (JNE), considerando como critério a familiaridade da palavra para uma criança entre 24 e 36 meses, e a representatividade da palavra com brinquedo/objeto, contemplando as Etapas 1 e 2. Os juízes crianças analisaram, em situação de coleta de dados, a familiaridade e a representatividade das palavras-estímulos, apresentado na Etapa 3. Após, realizou-se o estudo piloto- Etapa 4, com as palavras-estímulo selecionadas. Na análise estatística dos juízes especialistas e não especialistas utilizou-se o índice de Concordância do Kappa Fleiss e Gwet. Na análise das respostas dos juízes crianças e no estudo piloto, a análise foi feita em relação ao tipo de resposta da criança, especificamente pontuando a nomeação espontânea do brinquedo/objeto, pontuando de forma qualitativa.

**Resultados:**

O resultado da Etapa 1 e 2, e a concordância dos testes estatísticos para os critérios de Familiaridade e Representatividade foi de 45,7% para JE e 76,4% para JNE, e resultado de 100% para a concordância da Representatividade. Foram analisadas 122 palavras, resultantes dos critérios previamente estabelecidos, totalizando 34 palavras (exclusão de uma palavra por parte das pesquisadoras), totalizando 33 palavras-estímulos. Na Etapa 3, das 33 palavras-estímulos aplicadas, nove apresentaram pontuação de nomeação espontânea abaixo do esperado, sendo retestadas para a Etapa 4, Estudo Piloto. O resultado do Estudo Piloto evidenciou que das nove palavras-estímulos retestadas, quatro delas ainda apresentaram pontuação abaixo, sendo excluídas do estudo. Portanto com a aplicação do estudo piloto a lista expandida do PEEPS-BP, resultou em 29 palavras.

**Conclusão:**

O PEEPS-BP - Lista Expandida apresentou evidências de validade de conteúdo satisfatórias para a adaptação transcultural do teste.

## INTRODUÇÃO

A avaliação fonológica e lexical de crianças entre 24 e 36 meses geralmente é realizada por meio de amostra da fala espontânea. Dessa maneira, é necessário um exemplar significativo de dados para obter o perfil fonológico e o vocabulário de uma criança nessa faixa etária. Por isso, para crianças maiores de três anos há mais opções de instrumentos padronizados e disponibilizados na literatura para suprir essa demanda^(1-3)^.

O Instrumento *Profiles of Early Expressive Phonological Skills* (PEEPS) foi desenvolvido para avaliar o vocabulário e a fonologia de crianças entre 18 e 36 meses, referenciado como PEEPS-US (*United States*). O PEEPS-US^(3)^ possui duas listas de aplicação na avaliação: a Lista Básica para crianças de 18 a 24 meses, composta de 40 palavras-estímulos; e a Lista Expandida, para ser aplicada junto à Lista Básica, nas crianças a partir dos 24 até os 36 meses, sendo esta composta por mais 20 palavras-estímulos. O teste em questão, portanto, é dinamizado com o uso de brinquedos que representam as palavras-estímulos e aplicado em contexto lúdico.

Os estudos teóricos para a formação do PEEPS-US^(3)^ foram realizados a partir de palavras existentes no *MacArthur-Bates Communicative Development Inventories* ^(4)^ *(MacArthur-Bates - CDI)*, utilizando a versão de palavras e sentenças, de acordo com normativas de idade de aquisição do Inglês. Nesse contexto, adaptar um instrumento validado em outro país favorece o intercâmbio de informações científicas, possibilitando estudos transculturais e garantindo inferências e resultados que o teste pode proporcionar para a população a ser assistida^(5,6)^.

No Português Brasileiro, poucos são os instrumentos que fornecem componentes necessários para o desenvolvimento da linguagem em uma mesma avaliação, como vocabulário e fonologia, por exemplo^(7,8)^. Outrossim, a avaliação em crianças tão pequenas, na faixa etária anteriormente mencionada, também é restrita na literatura nacional. Assim, vale destacar que o teste ABFW^(9)^, reconhecido nacionalmente e de valiosa contribuição para a Fonoaudiologia do Brasil, apresenta componentes interligados em uma mesma testagem, disponibilizada por meio de figuras para o público a partir dos dois anos. Os dados referentes à validação desse instrumento para as Provas de Fonologia e de Vocabulário encontram-se nas pesquisas de doutoramento das autoras Wertzner^(10)^ e Befi-Lopes^(11)^.

Para o processo de validação da prova de fonologia foram realizados estudos teóricos a partir de instrumentos internacionais de avaliação da fala, para o delineamento de palavras-estímulos, realizando posterior análise de juízes para seleção das pranchas de ilustração. A autora apresenta dados normativos por faixa etária, além de análise de correlação para o método de coleta de imitação e nomeação, distribuindo os resultados para a produção dos fonemas nos diferentes grupos estudados^(10)^.

Na prova de vocabulário foi realizada a análise da competência e desempenho lexical em nove campos conceituais, além disso, a comparação em crianças com e sem alteração de fala/articulação. A análise apresenta os dados normativos para as diferentes classes dos processos de designações lexicais, distribuídas entre a amostra pesquisada^(11)^.

A adaptação transcultural do PEEPS-US^(3)^ para o Português Brasileiro, com validação de conteúdo da Lista Expandida, intitulado de PEEPS-BP (*Brazilian Portuguese*) - Lista Expandida, tem o intuito de desenvolver um instrumento que contribuirá para a avaliação precoce de crianças auxiliando, sobretudo, no diagnóstico e intervenção clínica, assim como na pesquisa científica.

O PEEPS-BP - Lista Expandida se trata da validação de conteúdo das palavras-estímulos para compor a lista expandida do instrumento no idioma nacional, com critérios pertinentes ao vocabulário e fonologia, o qual é dinamizado com crianças entre 24 e 36 meses. Com isso, tal etapa corresponde ao processo de verificar a adequabilidade para a realidade brasileira e a análise linguística (semântica e fonológica) dos itens que compõem o PEEPS-BP - Lista Expandida. Desse modo, o conteúdo é verificado a partir da análise de *expertises* da área, a fim de compreender os itens que compõem o instrumento, pontuando sua relevância e pertinência para a aplicabilidade no território brasileiro^(12,13,14,15)^.

O objetivo deste estudo foi buscar evidências de validade de conteúdo do PEEPS-BP - Lista Expandida, a ser aplicado junto à Lista Básica existente em crianças de 24 a 36 meses.

## MÉTODO

Esse estudo se trata de uma adaptação transcultural, quantitativo e transversal, considerando critérios psicométricos. A adaptabilidade do PEEPS-BP foi autorizada pelas autoras do PEEPS-US e elaborada em conjunto com as autoras da Lista Básica^(16)^ do PEEPS-BP antecessora à aplicação da Lista Expandida que é foco desse artigo.

Esta pesquisa foi devidamente aprovada pelo Comitê de Ética em Pesquisa, registrada com o número 18419319300005346. Foi solicitada a autorização de todos os participantes envolvidos, conforme as normas do Conselho Nacional de Saúde - Resolução 466/12. Todos os participantes foram submetidos à assinatura do Termo de Consentimento Livre e Esclarecido (TCLE). Além disso, é válido ressaltar que as crianças incluídas no estudo assentiram a sua participação na pesquisa de forma oral.

### Participantes e procedimentos

A amostra foi composta por diferentes participantes, os quais foram recrutados por conveniência, e/ou contatados via e-mail e/ou telefone pelas autoras. Esse público, então, foi captado e compôs uma das quatro etapas do processo de adaptação, quais sejam: Juízes Especialistas (Etapa 1), Juízes Não-Especialistas (Etapa 2), Juízes Crianças (Etapa 3) e Estudo Piloto (Etapa 4).

### Etapa 1 - juízes especialistas

Esta etapa, inicial no processo de adaptação do instrumento, teve como objetivo verificar quais palavras são familiares ao vocabulário de uma criança de 24 a 36 meses por meio do julgamento dos conceitos teóricos de *experts*. *A*inda, buscou-se definir, dessas palavras, que poderiam ser representadas por um brinquedo. Diante disso, profissionais foram contatados por e-mail e convidados a participar desta etapa da pesquisa. Todos que aceitaram participar assinaram o TCLE.

A amostra de Juízes Especialistas (JE) foi composta por nove profissionais, dentre eles fonoaudiólogos e linguistas com *expertise* na área de linguagem/fala, especialmente fonologia e vocabulário infantil, todos com doutorado.

Nesta Etapa 1, cada JE realizou individualmente sua análise por aproximadamente dois meses, tempo disponibilizado para que pudessem enviar as respostas. Para a análise, foi enviado um link que direcionava o JE para dois formulários do *Google Docs*. Ambos os formulários apresentavam as 423 palavras do Inventário de Desenvolvimento Comunicativo MacArthur do Português Brasileiro - palavras e sentenças^(17)^.

No primeiro formulário, cada JE deveria responder acerca da Familiaridade da palavra, ou seja, considerar se a palavra estaria presente no vocabulário infantil tanto receptivo como expressivo de uma criança a partir de 24 meses. Se, de alguma forma, as crianças nesta faixa etária já teriam sido expostas e estariam familiarizadas com a palavra. O julgamento das respostas foi realizado por meio da Escala Likert dividida em: Extremamente Familiar (1), Muito Familiar (2), Familiar (3), Pouco Familiar (4) e Não Familiar (5). O JE, deveriam classificar de acordo com a Escala, a proximidade do vocábulo para faixa etária pesquisada, julgando por exemplo, palavras consideradas frequentes no vocabulário, como Extremamente Familiar.

No segundo formulário, os JE responderam ao critério de Representatividade, ou seja, se a palavra analisada poderia ser representada por meio de um brinquedo. Nesse formulário a resposta deveria ser assinalada como ‘’Sim” ou ‘’Não”.

Concluída esta etapa, as respostas dos especialistas foram organizadas em uma tabela no programa Excel, para posterior análise estatística.

### Etapa 2 - juízes não-especialistas

Esta etapa apresenta os mesmos objetivos e critérios da etapa anterior (Etapa 1). No entanto, este grupo foi composto por mães de crianças entre 24 e 36 meses de idade, denominadas Juízes Não-Especialistas (JNE). Todas assinaram o TCLE concordando em participar da pesquisa.

A amostra de JNE foi composta por oito mães que não possuíam conhecimento técnico/específico na área de linguagem/fala e fonologia/vocabulário infantil, e que possuíam filhos(as) na faixa etária do estudo. Buscou-se verificar quais palavras estão presentes no vocabulário de crianças pequenas a partir do olhar criterioso destas que convivem cotidianamente com os infantes.

Da mesma forma que os JE, os JNE deveriam responder aos dois formulários do *Google Docs*, disponibilizados no formato virtual/on-line e com 423 palavras do Inventário de Desenvolvimento Comunicativo MacArthur do Português Brasileiro- palavras e sentenças^(17)^.

A análise dos JNE também considerou os mesmos critérios para Familiaridade e Representatividade. Na familiaridade solicitou-se que avaliassem se a palavra pertencia ao vocabulário de uma criança de 24-36 meses, indicando se a palavra era: (1) Extremamente Familiar, (2) Muito Familiar, (3) Familiar, (4) Pouco Familiar e (5) Não Familiar. Em relação à Representatividade, as JNE precisavam apontar se consideravam que a palavra poderia ser representada por um brinquedo, respondendo “Sim” ou “Não”.

Assim como na Etapa 1, ao final as respostas dos JNE foram organizadas em tabela no programa Excel, e submetidas à análise estatística. Assim sendo, tanto na Etapa 1 quanto na Etapa 2, a análise foi realizada transversalmente do método estatístico, com a finalidade de quantificar o índice de concordância entre os JE e JNE em relação à acurácia das palavras.

Nessa ótica, o índice de concordância do Kappa Fleiss, responsável por gerar a função discriminante dos dados do teste e a linha de corte da concordância dos juízes nas 423 palavras, foi utilizado para o critério de Familiaridade, ao passo que o coeficiente de *Gwet* foi utilizado para analisar o critério da Representatividade das palavras, considerando o valor absoluto para essa análise. Após, foi realizada a interseção dos conjuntos para encontrar palavras que apresentaram índice de concordância superior na Familiaridade e valor absoluto no coeficiente de *Gwet* para a Representatividade.

Nesse cenário, o índice de concordância de 1 a 1,5 (17,8%) das palavras foi o primeiro a ser analisado pelo critério, classificando-as como “Extremamente Familiar”. Essas palavras apresentaram a concordância na interseção Familiaridade e Representatividade. Este foi considerado o ponto de corte principal, totalizando 42 palavras na categoria.

Posteriormente, analisou-se o ponto de corte na sequência entre 1,5 - 2,5 para abranger um número maior de palavras, que apresentassem a mesma proposta da interseção Familiaridade e Representatividade. Foram incluídas mais 200 palavras, os quais foram acrescidos ao ponto de corte inicial de, 1-1,5.

Por fim, com a última análise empreendida optou-se por formar a lista de palavras da pesquisa, apresentada na seção resultados, que compreendeu o ponto de corte entre 1 e 1,5-2. Esse ponto de corte apresenta as palavras relacionadas à interseção da Representatividade e concordância com o critério de Familiaridade nos eixos “extremamente familiar” e “muito familiar”.

Definido este ponto de corte, as palavras foram analisadas na correspondência de 1-1,5 até 2. As palavras presentes nesse conjunto, totalizaram 122 e foram as que obtiveram pontuação significativa e relevante na interseção entre Familiaridade e Representatividade. Essas 122 palavras foram analisadas, ainda na Etapa 2, conforme os critérios a seguir:

- Palavras presentes no instrumento original passíveis de tradução e adaptação adequada para o PB;
- Prioridade para as primeiras palavras obtidas na classificação da análise estática (42 palavras) que obtiveram o melhor escore analisado a partir das respostas dos juízes (<1,5), as quais são da categoria “Extremamente Familiares”.
- Palavras com estrutura silábica formadas por dissílabos, seguido de palavras trissílabas que contemplassem fonemas esperados para a idade de aquisição fonológica do estudo (24 a 36 meses).

Finalizadas as Etapas 1 e 2 de seleção das palavras a serem inseridas no instrumento, seguiu-se para as etapas de validação de conteúdo e aplicação do PEEPS-BP- Lista Expandida.

### Etapa 3 - juízes crianças

A Etapa 3 teve como objetivos verificar se as palavras selecionadas (Etapas 1 e 2) faziam parte do vocabulário de crianças pequenas, bem como se os brinquedos selecionados eram adequados para reconhecimento e nomeação espontânea por crianças de 24 a 36 meses. Para tanto, as autoras da pesquisa adquiriram diversos brinquedos disponibilizados comercialmente, para aplicação do instrumento de acordo com a lista final de palavras oriundas das Etapas 1 e 2.

A amostra desta Etapa 3 foi constituída por quatro crianças selecionadas por conveniência, com idades entre 24 e 36 meses, especificamente duas do sexo masculino e duas do feminino. Os pais/responsáveis consentiram com a participação na pesquisa, e as crianças concordaram oralmente em integrar o estudo.

Nesta etapa foram realizadas avaliações fonoaudiológicas para assegurar o desenvolvimento cognitivo, linguístico, auditivo e motor das crianças dentro do esperado para a etapa em que se encontravam. Com isso, apenas seriam excluídas da pesquisa aquelas que apresentassem comprometimento no neurodesenvolvimento, sistema auditivo, linguagem oral, receptiva e/ou expressiva. Com os resultados obtidos, nenhum participante foi excluído.

As avaliações descritas a seguir foram aplicadas a cada participante individualmente:

- Anamnese Geral - foi realizada uma entrevista online, a partir da plataforma *Google Meet*, para recrutar informações sobre o período gestacional, complicações pré-peri e pós-natais, assim como fatores relacionados ao desenvolvimento infantil, à saúde geral, às situações de vida cotidiana e social relacionados à saúde e à família.
- Avaliação Auditiva - foi realizada com o aparelho de Emissões Otoacústicas Transientes (EOAT) da marca OTOREAD. Nesse exame a criança deveria apresentar a resposta “passou” em ambas as orelhas, caracterizando função coclear adequada.
- Escala Bayley de Desenvolvimento Infantil III (Triagem)^(18)^ avalia três domínios do desenvolvimento: cognitivo, linguístico (comunicação expressiva e receptiva), motor (amplo e fino). A criança deveria apresentar como resposta “competente” nos quatro níveis da escala, de acordo com sua faixa etária. Importante ressaltar que a pesquisadora responsável possui formação para a aplicação desse instrumento.
  - Inventário MacArthur do Português Brasileiro - palavras e sentenças^(17)^: os pais/responsáveis, deveriam pontuar no protocolo do inventário se a criança produzia, no mínimo, 10 palavras das previstas no documento, ou seja, a criança precisava expressar ao menos 10 palavras. Trata-se de um marcador avaliativo de nível mínimo de resposta de produção da fala.

Assim sendo, de acordo com os testes aplicados, as quatro crianças foram incluídas na pesquisa, caracterizando desenvolvimento neurotípico para a faixa etária selecionada.

Os procedimentos da Etapa 3 foram realizados em sala apropriada para coleta de dados, ou seja, obter as respostas das crianças frente aos estímulos apresentados. Os pais/responsáveis acompanharam todas as etapas da pesquisa feita com a criança. Além disso, a coleta de dados foi gravada e filmada para posterior análise de dados, processo consentido pelos pais, e de acordo com o Termo de Confidencialidade da pesquisa.

Em uma sala, devidamente estruturada com tatames infantis, a pesquisadora disponibilizou os brinquedos que representavam as palavras-estímulo, que estavam em uma sacola transparente para a análise da criança. Em um mesmo brinquedo pode haver a possibilidade de nomeação de outras palavras-estímulos do instrumento, como por exemplo, as partes do corpo em uma boneca. A instrução fornecida pela pesquisa foi traduzida do idioma original (Inglês) para o PB, compondo a seguinte frase:

“Olá, eu vou mostrar para você alguns brinquedos, você deve me falar o nome de cada brinquedo e se conhece eles. No final, você poderá brincar livremente com todos os brinquedos junto à mamãe/responsável”.

Após a instrução, a criança escolhia os brinquedos conforme seu interesse, já que estavam visíveis na sacola, e então era instigada a realizar a nomeação espontânea, identificando o brinquedo. Ainda, o instrumento original permite o uso de estratégias de estímulo conforme a necessidade, que deve ser realizado utilizando perguntas como “*O que é isso?*” ou “*Fale para a mamãe o nome desse brinquedo*”, bem como completando alguma frase ou utilizando o contexto de repetição.

Na Etapa 3 o objetivo era apenas a nomeação espontânea das crianças a partir dos objetos apresentados, portanto, a coleta foi conduzida de acordo com o interesse da criança em manipular a sacola transparente. Esta forma de aplicação posteriormente foi modificada na Etapa 4, pela separação dos objetivos em categorias, a fim de não dispersar a criança com todos os brinquedos expostos.

Os dados dessa etapa foram analisados, pontuando se houve a nomeação espontânea por parte da criança, ou se ela realizava a repetição do vocábulo fornecido pela pesquisadora ou, ainda, se não sabia falar. Também foram pontuadas as situações em que a criança sabia a palavra, porém ficou em dúvida em relação à representatividade do brinquedo. Nesse ínterim, na Etapa 3 as respostas das crianças foram pontuadas com 1 ponto para cada palavra produzida pelas crianças no critério “nomeação espontânea” e 0 ponto para “repetição ou recusa” ao falar. Também, foi analisado qualitativamente quando elas apresentavam reações do tipo não saber o que era o brinquedo ou trocavam por outro nome, mostrando a dúvida sobre a representatividade do item. A soma das pontuações das quatro crianças deveria ser superior a três para considerar a palavra como, Adequada para a representatividade do brinquedo e Adequada para a Familiaridade da palavra.

### Etapa 4 - estudo piloto

Na Etapa 4, Estudo Piloto (EP), o objetivo foi de administrar o PEEPS-BP- Lista Expandida em uma situação real de coleta, a partir das modificações observadas na Etapa 3.

Foram recrutadas para o EP, por conveniência, seis crianças com idades entre 24 e 36 meses, sendo três do sexo masculino e três do feminino. Os pais/responsáveis assentiram com o TCLE e as crianças concordaram em participar da pesquisa de forma oral. Estas crianças foram submetidas aos mesmos instrumentos da etapa anterior (Etapa 3) para a inclusão nesta etapa do estudo, ou seja, a fim de confirmar seu desenvolvimento infantil dentro dos parâmetros esperados para a faixa etária. Portanto, foram realizados os procedimentos de anamnese geral, Avaliação Auditiva por EOAT, Escala Bayley de Desenvolvimento Infantil III (Triagem)^(18)^ e avaliação do Inventário de Desenvolvimento Comunicativo MacArthur do Português Brasileiro - palavras e sentenças^(17)^.

As palavras-estímulos estavam representadas pelos brinquedos e foram previamente selecionadas de acordo com os resultados da Etapa 3. A partir disso, os objetos foram colocados em sacolas transparentes menores e dentro de caixas individuais, agrupados de acordo com a categoria semântica, ou contexto mais próximo, para favorecer a interação. O agrupamento dos brinquedos se adequou às categorias de animais, de utensílios de cozinha e comida, de boneca com as partes do corpo, e utensílios de casa com veículos e de brinquedos e miscelâneas.

Ao iniciar a aplicação do Instrumento PEEPS-BP - Lista Expandida, a pesquisadora forneceu a instrução inicial, traduzida do PEEPS-US^(3)^ e adaptada para o novo formato de coleta do EP: “*Eu vou mostrar para você os brinquedos que estão nessas caixas, você deve me falar o nome de cada brinquedo e se conhece eles. Quando terminarmos de ver todas as caixas com os brinquedos, você poderá brincar com todos eles e com a mãe/pai/responsável*”.

Os brinquedos, divididos nas categorias acima expostas, foram entregues para a criança, assegurando que fossem escolhidos aleatoriamente, conforme execução do instrumento original. Em tal contexto e com o intuito de auxiliar a nomeação espontânea, quando necessário foram utilizadas estratégias com perguntas para facilitar a pronúncia da palavra, como por exemplo “*O que é isso?*” ou “*Fale para a mamãe o nome desse brinquedo*”, assim como completando alguma frase ou sua repetição. A aplicação do EP foi gravada e filmada conforme critérios do Termo de Confidencialidade, para registrar as informações de dados da pesquisa e posterior análise.

Nesta Etapa 4 (EP) analisou-se a aplicação do Instrumento PEEPS-BP- Lista Expandida com o formato estabelecido de brinquedos dispostos por categorias semânticas e, ainda, realizou-se a análise qualitativa dos exemplares modificados em relação ao resultado da Etapa 3. No EP, não foram considerados a repetição da palavra, somente a nomeação espontânea. Com isso, os itens testados deveriam apresentar pontuação mínima de cinco pontos para permanência no estudo, ou seja, mesmo critério da Etapa 3.

A nomeação espontânea considerada em todo o método do estudo, diz respeito a identificação e reconhecimentos adequados da palavra-estímulo diante do objeto/brinquedo, não sendo caracterizado por uma repetição/modelo fornecido pelo pesquisador. Essa questão implica em considerar na nomeação espontânea, possíveis omissões/substituições fonológicas nas palavras-estímulos, visto que, o sistema fonológico da criança está em período de aquisição e desenvolvimento fonológico. Ou seja, na faixa etária do estudo, a maioria das crianças em aquisição típica não adquiriram a estrutura CCV; portanto, se a criança nomeasse “falda” [‘faw.da] para “fralda” [‘fraw.da], considerava-se que realizou a nomeação espontânea do objeto.

O Quadro 1 descreve a sequência metodológica utilizada na pesquisa científica, indicando os participantes e a escolaridade.

**Quadro 1:** Etapas metodológicas da pesquisa

Validação de Conteúdo	Número Amostral	Critérios de Seleção	Escolaridade dos participantes
Etapa 1-Juízes Especialistas	9 Juízes (6 Fonoaudiólogos e 3 Linguistas)	Fonoaudiólogos ênfase em linguagem/fala, especialmente fonológica e vocabulário Linguistas ênfase em fonologia.	Pós-graduação em nível de doutorado
Etapa 2-Juízes Não especialistas	7 Juízes	Mães ‘’leigas” de crianças entre 24 e 36 meses, ou seja, sem conhecimento teórico científico sobre desenvolvimento dos aspectos da linguagem oral.	4 mães com Ensino Superior Completo
			3 mães com Ensino Médio Completo
Etapa 3-Juízes Crianças	4 crianças sexo feminino (29 e 36 meses) e sexo masculino (27 e 31 meses)	Crianças entre 24 e 36 meses, duas do sexo masculino (27 e 31 meses) e duas do sexo feminino (29 e 36 meses), desenvolvimento típico cognitivo, motor amplo e fino, linguagem expressiva e compreensiva. (Conforme avaliações realizadas)	3 mães com Ensino Superior Completo
			1 mãe com Ensino Médio Completo
			4 crianças matriculadas no Maternal I
Etapa 4-Estudo Piloto	6 crianças sexo feminino (32,33 e 34 meses) e sexo masculino (26,35 e 36 meses)	Crianças entre 24 e 36 meses, três do sexo masculino e três do sexo feminino, desenvolvimento típico cognitivo, motor amplo e fino, linguagem expressiva e compreensiva. (Conforme avaliações realizadas)	6 mães com Ensino Superior Completo
			3 crianças matriculadas no Maternal I e II
			3 crianças não matriculadas em escolas.

## RESULTADOS

Os resultados das Etapa 1 e 2 culminaram em 122 palavras, conforme os índices de concordância aplicados, resultado do ponto de corte de análise de 1-1,5-2. Das 122 palavras selecionadas, conforme os critérios estabelecidos anteriormente na seção do Método - Etapa 2, Itens a-b-c, foram selecionadas 34 palavras. No entanto, uma palavra foi previamente excluída: “*menino/menina*”, devido a flexão do morfema que indica a flexão de gênero o/a. Sendo assim, o resultado das Etapas 1 e 2 totalizou 33 palavras.

O resultado da concordância entre os JE e JNE, para o coeficiente Kappa Fleiss em relação ao coeficiente de Gwet, apresentou valor igual a 45,7%, com intervalo de confiança de 95% para os JE e 76,4% para as JNE, com mesmo intervalo de confiança considerado. Em relação apenas à análise da Representatividade, o índice de concordância de Gwet, para JE e JNE apresentou resultado absoluto de 100%.

Observou-se que na Etapa 3, nove palavras-estímulos analisadas qualitativamente em relação a nomeação espontânea e/ou reconhecimento do brinquedo, apresentaram pontuação inferior ao esperado na aplicação do instrumento para crianças juízes. Sendo assim, optou-se por realizar o reteste dos brinquedos/objetos na Etapa 4.

Na Etapa 4 do EP, os resultados das palavras-estímulos analisadas qualitativamente, e que ainda apresentaram pontuação inferior ao esperado (cinco pontos) foram excluídas do instrumento. Assim, das 33 palavras testadas, o PEEPS-BP - Lista Expandida ficou constituído por 29 palavras-estímulo para avaliação de crianças de 24 a 36 meses.

O resultado da proposta de aplicação foi modificado na Etapa 4 em relação à Etapa 3, sem causar prejuízo quando comparado ao instrumento original, pois tratava-se ainda de uma análise dos Juízes Crianças para o reconhecimento dos estímulos. Porquanto manteve-se a entrega aleatória dos brinquedos, mesmo que tenham sido agrupados em categorias semânticas. A vista disso, o cenário da coleta de dados foi satisfatório para a execução do EP, de maneira a manter a sequência para os demais estudos.

O Quadro 2 apresenta a sequência dos resultados por etapas (Etapas 1, 2, 3 e 4) das palavras-estímulos.

**Quadro 2:** Resultados das Etapas 1, 2, 3 e 4 para as palavras-estímulos

ETAPA	NÚMERO DE PALAVRAS	PALAVRAS	PALAVRAS ABAIXO DA MÉDIA	PALAVRAS EXCLUÍDAS
Etapa 1 e 2 - JE e JNE	34 palavras	Banana, balão, barriga, boca, boneca, cabelo, caminhão, cobertor, colher, cachorro, cama, chapéu, copo, dente, elefante, fralda, lua, leão, língua, mamadeira, mão, meia, neném, orelha, passarinho, pé, peixe, perna, pente, suco, sol, sabonete, umbigo, menino/menina	Para essa etapa não foram consideradas palavras abaixo da média.	Menino/menina
Etapa 3 - Crianças	33 palavras	Banana, balão, barriga, boca, boneca, cabelo, caminhão, cobertor, colher, cachorro, cama, chapéu, copo, dente, elefante, fralda, lua, leão, língua, mamadeira, mão, meia, neném, orelha, passarinho, pé, peixe, perna, pente, suco, sol, sabonete, umbigo.	Caminhão, cobertor, lua, meia, neném, passarinho, pente, perna e umbigo. (Estímulos retestados na Etapa 4)	Nesta etapa não foram excluídas palavras
Etapa 4 - Estudo Piloto	29 palavras	Balão, banana, barriga, boca, boneca, cabelo, cachorro, cama, chapéu, colher, copo, dente, elefante, fralda, leão, língua, mamadeira, mão, orelha, pé, peixe, sabonete, sol, suco, caminhão, lua, meia, pente e perna.	Para essa etapa não foram consideradas palavras abaixo da média.	neném, cobertor, passarinho e umbigo

## DISCUSSÃO

Várias etapas são necessárias para realizar a adaptação transcultural de um instrumento de avaliação, sendo a validação de conteúdo das palavras o passo inicial nesse processo^(19)^. Nesse contexto, é necessário pontuar que o estudo das palavras, dos elementos do instrumento original, das escolhas dos juízes/*expertises* a participarem do processo são determinantes para o conteúdo final^(20,21)^.

Em literatura aponta-se como possibilidade o uso de tradução e retrotradução quando se realiza a adaptação transcultural, mas tal prática não foi passível de ser realizada neste estudo pelo fato de a aquisição da fonologia, inventário fonético, do sistema vocálico e das estruturas silábicas, da língua Inglesa serem diferente do Português Brasileiro^(22,23)^. Ainda assim, foi utilizado o mesmo perfil do estudo teórico apresentado no instrumento original, ou seja, o Inventário MacArthur do Português Brasileiro- palavras e sentenças^(17)^, já adaptado para o Português Brasileiro. Este inventário compreende uma lista de palavras pertencentes ao vocabulário infantil, de modo que funcionou como base para a seleção das palavras-estímulos. Além disso, as escolhas das palavras-estímulos, dos juízes e dos critérios envolvidos foram definitivas para a elaboração adequada da lista expandida, uma vez que esses procedimentos auxiliam na validação de conteúdo para a adaptação de um instrumento, conforme pesquisadores da área apresentam em seus estudos^(24-26)^.

Ademais, a validade de conteúdo compreende a avaliação por parte dos juízes especialistas da área, os quais enfatizaram seu conhecimento teórico e científico sobre o desenvolvimento infantil, bem como acerca dos aspectos de fonologia e vocabulário, durante o processo de construção do estudo ora exposto. Também se notou tal fato com relação aos juízes não-especialistas, que, apesar de não apresentarem domínio teórico sobre os componentes da linguagem oral, apresentam convívio diário com crianças de 24-36 meses. Com base em estudos acadêmicos, sabe-se que a aquisição dos sons e das palavras, em parte, depende dos estímulos ambientais ofertados^(27)^, então, essa heterogeneidade dos juízes não-especialistas mostrou-se importante para o julgamento das palavras-estímulos, incluindo diferentes termos que integram o convívio e o contexto social de cada interação das crianças^(28-30)^.

O julgamento por parte das crianças, auxiliou a verificar os itens preliminares do instrumento, as palavras-estímulos, além de apontar brinquedos não satisfatórios para a representação do item. Observou-se qualitativamente, na condução do procedimento de pesquisa, que muitas vezes a criança apresentava em seu vocabulário a palavra, porém, o objeto confundiu ou não representou adequadamente o que estava sendo proposto. Por isso, o fato de sempre manter a interseção na análise estatística entre familiaridade e representatividade da palavra-estímulo foi uma constante no estudo, auxiliando na escolha do objeto/brinquedo, reforçando a escolha das palavras adequadas para o vocabulário infantil.

Os estudos de avaliação fonológica, ou de avaliação do vocabulário, em sua maioria utilizam figuras ilustrativas como componente de conteúdo, diferente do proposto para a seleção das palavra-estímulos para esse conteúdo^(9,31,32)^. Tal realidade é diferente do que acontece no PEEPS-BP- Lista Expandida, posto que o brinquedo para representar a palavra deve ser julgado pelo juiz não-especialista, mesmo que foi previamente julgado por juízes especialistas. Ainda, os juízes crianças são indispensáveis para esse tipo de validação, uma vez que essa população-alvo será a avaliada e beneficiada com os objetivos da aplicação do instrumento, sendo um critério sugerido na parte psicométrica^(33)^. Torna-se válido ressaltar, que o instrumento PEEPS-US e sua adaptação, é aplicado, sempre, com o uso de brinquedos/objetos.

Portanto, a avaliação por parte dos juízes envolvidos nesse processo compreendeu a validação de conteúdo e o desenvolvimento do mesmo com procedimentos quantitativos e qualitativos. Com isso, um instrumento é válido em seu conteúdo quando realmente avalia o objetivo proposto, que neste caso foi o de selecionar palavras familiares para o vocabulário de uma criança de 24-36 meses e que, ainda, fossem representadas por um brinquedo. Tal prática se deu devido à metodologia do instrumento original, que se realiza com objetos concretos.

À vista disso, os juízes cumpriram com o proposto de pontuar os estímulos do instrumento, garantindo que o instrumento representasse adequadamente o objetivo de ser avaliado. Com o resultado da estatística em relação à familiaridade da palavra, critérios teóricos foram estabelecidos a fim de compreender o componente fonológico e a estrutura silábica de acordo com a aquisição da faixa etária de 24-36 meses. No entanto, por se tratar de um instrumento que avalia vocabulário e fonologia concomitante em uma população infantil, algumas palavras que entraram no estudo apresentaram estrutura silábica e fonológica esperadas para domínio de aquisição após os 36 meses. Isso justifica pela palavra pertencer ao vocabulário infantil, de modo que já se sabendo que a criança poderia apresentar algum tipo de omissão fonológica, considerado comum para a idade de aquisição.

Por fim, justifica-se tal escolha pois testes que envolvem avaliações precisam incluir palavras-estímulos de complexidade variada, uma vez que assim fornecem respostas variadas e fidedignas acerca da capacidade da criança em situações fora do seu cotidiano^(13,14)^.

O PEEPS-BP- Lista Expandida, apresenta palavras-estímulos adequadas para o vocabulário de crianças de 24-36 meses, as quais contemplaram os critérios que, anteriormente, foram descritos no Método. O uso desses critérios previamente estabelecidos, evidenciam a efetividade da tradução e adaptação transcultural do instrumento para o Português Brasileiro, uma vez que, foram escolhidas as palavras com melhor escore, em diferentes contextos fonológicos e estruturas silábicas.

As palavras-estímulos apresentam tanto os fonemas da fase de aquisição da população estudada, quanto outros fonemas que devem ser adquiridos em faixa etária posterior, realizando o balanceamento fonológico variado e com diferentes níveis de complexidade.

Diante desse fato, justifica-se as palavras escolhidas para o instrumento PEEPS-BP - Lista Expandida apresenta: em Onset inicial (OI) os fonemas /p/, /b/, /d/, /k/, /m/, /s/ e /ʃ/; em Onset medial (OM) apresenta fonemas, /p/, /b/, /t/, /d/, /k/, /g/, /m/, /n/, /ɲ/, /ʃ/, /ɾ/, /l/, /x/, /ʎ/, alofone [tʃ]. Na posição da Coda medial (CM), apresenta o fonema /l/, os arquifonemas /N/, e /R/, e na posição de Coda final (CF) o fonema /l/ e arquifonema /R/. E no Onset Complexo (OC), apresenta o segmento /fɾ/ em OI. O PEEPS-BP-Lista Expandida, apresenta três palavras monossílabas, 16 palavras dissílabas, sete palavras trissílabas e três polissílabas.

A lista expandida, diferencia-se da lista básica, por apresentar alguns fonemas não contemplados na lista básica, apresenta segmentos de aquisição consonantal considerados tardios para a idade em questão avaliada. Ou seja, apresenta componentes que a lista básica não possui, valorizando sua aplicação nas crianças entre 24-36 meses, considerado o vocabulário destinado a elas.

Outrossim, a aplicação do EP é caracterizado como uma escala dos procedimentos, materiais e métodos propostos na aplicação do instrumento, ou seja, trata-se de uma versão do estudo completo que envolve tudo o que foi previsto na metodologia, de modo a possibilitar alterações ou melhorias do instrumento nas fases que antecedem a investigação de critério e construto^(15)^. Assim, a importância de conduzir um estudo piloto está na possibilidade de revisar, testar, aprimorar e avaliar o cenário de coleta, bem como as instruções do instrumento e o procedimento da pesquisa. O EP é capaz de fornecer problemas em potencial na pesquisa, para que sejam implementados antes de conduzir o número amostral estabelecido para o estudo.

Logo, o EP aqui apresentado foi administrado para crianças com desenvolvimento típico em um cenário real de coleta, de modo a ser realizado o mais próximo possível do instrumento original. Com isso, as crianças foram expostas aos brinquedos aleatoriamente, os quais estavam separados nas categorias semânticas adotadas. Nesse contexto, as crianças não apresentaram dificuldade em reconhecer e produzir a palavra-estímulo a partir do brinquedo, apresentando um desempenho favorável em relação ao vocabulário e à fonologia. Esse formato foi estabelecido, ao separar por categorias semânticas e ser escolhido aleatoriamente pela criança, conforme o instrumento original, pois observou-se na etapa 3 que elas poderiam dispersar-se com o procedimento adotado.

A adaptação transcultural com a validade de conteúdo cumpriu os requisitos psicométricos para a escolha das palavras-estímulos com os diferentes juízes adotados, alcançando o objetivo de encontrar palavras familiares a crianças de 24 a 36 meses e que fossem possíveis de serem representadas por um brinquedo/objeto concreto. Ademais, o reforço da aplicação em um estudo piloto possibilitou identificar falhas e verificar a aplicabilidade do instrumento PEEPS-BP- Lista Expandida.

Nesse sentido, a validação de conteúdo sendo uma das etapas pertinentes da adaptação transcultural, apresentou a formação do PEEPS-BP Lista Expandida, para o contexto brasileiro. Para seguimento de outras medidas psicométricas, necessita-se ainda de pesquisa da validação de critérios e dados normativos para a população de estudo.

## CONCLUSÃO

O PEEPS-BP - Lista Expandida, adaptado para o Português Brasileiro, é considerado um instrumento de avaliação do vocabulário e fonologia infantil de crianças de 24 a 36 meses. Sua adaptação transcultural apresentou a validação de conteúdo das palavras, incluindo análise de diferentes juízes e seguindo a proposta do instrumento original possibilitará a avaliação de crianças pequenas. A avaliação do PEEPS-BP considerando sua aplicação nos anos iniciais da população infantil, favorecerá o acompanhamento e monitoramento do período de desenvolvimento destinado a linguagem oral, nos aspectos do vocabulário e da fonologia. A validação de conteúdo apresenta os itens adequados para a realidade infantil brasileira, assim como a diferença na aplicação do instrumento com objetos/brinquedos, favorecendo a interação entre profissional e criança, com o lúdico no momento avaliativo.

Dessa forma, conclui-se que o instrumento PEEPS-BP- Lista Expandida, poderá auxiliar na clínica fonoaudiológica, bem como na pesquisa, no acompanhamento dos marcos do desenvolvimento da linguagem oral assim como, a prevenção e promoção dos marcos do desenvolvimento da primeira infância, no que concerne aos aspectos da expressividade da fonologia e vocabulário. Sabe-se da importância de dar continuidade ao estudo, buscando critérios normativos, a partir da Lista Expandida estabelecida.
